# A mindful approach with end-of-life thoughts

**DOI:** 10.3389/fpsyg.2014.00138

**Published:** 2014-02-21

**Authors:** Francesco Pagnini, Deborah Phillips, Ellen Langer

**Affiliations:** ^1^Department of Psychology, Catholic University of MilanMilan, Italy; ^2^Department of Psychology, Harvard UniversityCambridge, MA, USA; ^3^Niguarda Ca' Granda HospitalMilan, Italy

**Keywords:** suicidal ideation, amyotrophic lateral sclerosis (ALS), mindfulness, clinical psychology, end-of-life issues

Stutzki et al. (2012) reported interesting results about the attitudes toward assisted suicide and life-prolonging measures of people with Amyotrophic Lateral Sclerosis in a Swiss sample. Exploring these attitudes may be of particular interest given that assisted suicide is legal in Switzerland and people with severe chronic diseases can, with the support of a physician, actively decide to end their life.

While the relatively low percentage of subjects in the study who expressed intention to hasten death seems surprising, more than a third reported thinking about committing suicide. Moreover, clinical experience suggests that the sample's high non-participation rate may hide a more significant presence of suicidal thoughts in the ALS population. The meaning of this choice is different for each individual, merging its ethical, clinical, scientific, spiritual, social and legal aspects and making it a highly complicated challenge (Pagnini, 2013). The certainty underlying of beliefs and attitudes of patients, relatives, physicians and social workers is tested by such choice, and the severity of the decision makes it an extremely important clinical issue for physicians and researchers.

Without discussing the secularized right to commit suicide, which is outside the purview of this commentary, a clinical comment can be made about the motivations that sometimes push the patient to commit suicide. Psychological factors such as depression, hopelessness, loss of meaning and low existential well-being have been reported to be associated with a life-ending desire. Even if the physician is positively disposed about the potential for assisted suicide, if the wish is grounded in depression and hopelessness it appears inappropriate to see it as a clinical success. Ending a life may be seen in these cases as the only way to end the suffering. Most of the time, however, suicide is not the only option to stop or at least reduce suffering. The care providers also have an important role in the management of the patient's health, including both physical and psychological well-being, which directly influences suicidal thoughts.

One of the concepts that we believe can promote better clinical practice is mindfulness. Mindfulness, as we envisioned in the 1970s, can be seen as a mindset with one's cognitions focused on being aware of novelty in experiences or situations, perceiving subtle as well as large differences in contexts and events from which one derives a focus on the present and an avoidance of judgment (Langer, 1989). It is the reverse of mindlessness, in which the mind is trapped in a rigid mindset and unaware of individual and contextual changes, considering only a single perspective, relying on automatic or repetitive thought processes, judgments and behavior. In medicine, a common example of mindless attitude is defining a person by the diagnosis, ignoring or not accounting for the individual as a non-diagnosed person. A mindful clinical practice could help find different ways to see the patient's situation and self that would have a positive effect of the patient's psychological well-being. Many years of research about chronic disorders suggest that a mindful attitude on the part of patient, physician and the social environment, reduces the negative psychological impact of the illness (Phillips and Pagnini, 2014). Developing a mindful attitude can be highly effective in mediating some of the debilitating physical and psychological impacts of chronic disease that lead to suicidal wishes.

Moreover, as suggested by the authors, health professionals should develop an openness and willingness to discuss suicide with patients, without prejudice, and in a mindful way. A positive clinical relationship is required to understand the patient's real needs. Clinical experience suggests that behind the question “please, help me end my life” there are different needs, with different solutions (Olney and Lomen-Hoerth, 2005). If the care provider mindlessly considers this question, without pursuing deeper questioning to gain greater perspective and understanding, the proposed solution may well respond to an expressed hopelessness but not fulfill the patient's unmet needs. For example, one possible need underlying a suicidal request is the wish to stop being a burden to one's caregivers; addressing this question may lead to a different solution than solely addressing the suicidal request. Clinicians need to discriminate between these questions, that is, to allow for the possibility that the expressed wish to hasten death may be an expression of a different need, and to provide the patient with the clinical space to discuss it (Albert et al., 2005). Similarly, a patient discussing suicide with a physician who perceives it as an act against nature may be a heavily frustrating experience for the patient who may need to see that option included in a set of alternatives even if the physician is unwilling to comply with such a request. Prejudices, as any other rigid categories that influence our reasoning, thwart helpful discussion. The importance of this topic requests an individualized approach that cannot be generalized, reinforcing the criticality of a physician's self-awareness of pre-conceived assumptions and biases.

The complexity of the topic and its multiple implications make the definition of specific guidelines difficult at best and often likely to be unhelpful. A mindful person-centered approach, instead of a mindless patient-centered one could dramatically increase the quality of care particularly in such traumatic situations. High-quality care allows the person to explore more options to meet challenges related to the disease, and may come to see that suicide may not be the only or the best way to do so.
